# The Born Rule—100 Years Ago and Today

**DOI:** 10.3390/e27040415

**Published:** 2025-04-11

**Authors:** Arnold Neumaier

**Affiliations:** Fakultät für Mathematik, Universität Wien, Oskar-Morgenstern-Platz 1, A-1090 Wien, Austria; arnold.neumaier@univie.ac.at

**Keywords:** Born rule, probability in quantum mechanics, statistical interpretation of quantum mechanics, 81-03, 81P10, 81P15

## Abstract

The details of the contents and formulations of the Born rule have changed considerably from its inception by Born in 1926 to the present day. This paper traces the early history of the Born rule 100 years ago, its generalization (essential for today’s quantum optics and quantum information theory) to POVMs around 50 years ago, and a modern derivation from an intuitive definition of the notion of a quantum detector. Also discussed is the extent to which the various forms of the Born rule have, like any other statement in physics, a restricted domain of validity, which leads to problems when applied outside this domain.

## 1. Ten Formulations of the Born Rule

In today’s quantum mechanics textbooks, the Born rule, in one or the other of its many forms, is taken to be the axiomatic cornerstone of Max Born’s statistical interpretation of quantum mechanics, conceived in 1926. At that time, an unspecific probabilistic interpretation of quantum mechanics was already in the air.

For example, we read in Jeans ([1], p. 91) (1924) *“Obviously quantum-dynamics must be intimately concerned with the probability of such jumps. […] measure, in some way at present unknown, the probabilities of jumps in the velocity and perhaps also in the position of an electron which forms part of an atomic system.”* And in Bohr et al. ([2], p. 75) (1924), we read *“In addition, we assume that the occurrence of transition processes, both for the given atom itself and for the other atoms, with which it communicates, is linked with this mechanism through probability laws that are analogous to the laws of Einstein’s theory for the transitions induced by external radiation between stationary states.”.*

(German original: *“Ferner nehmen wir an, dass das Vorkommen von Übergangsprozessen, sowohl für das gegebene Atom selbst wie für die anderen Atome, mit denen es kommuniziert, mit diesem Mechanismus durch Wahrscheinlichkeitsgesetze verknüpft ist, die den Gesetzen der Einsteinschen Theorie für die von äusserer Strahlung induzierten Übergänge zwischen stationären Zuständen analog sind.”*).

In February 1926, this interpretation was already semiquantitative, and valid for large quantum numbers: *“According to the quantum theory, the inner state of an atom can change only in sudden jumps. Only the discrete stationary states of the atoms are possible. In place of the classical continuous absorption of energy we therefore have a discontinuous one in single jumps, for which certain transition probabilities exist. Between both must then exist correspondence relations, i.e., an approximate, for large quantum numbers even exact, agreement between the classical and the quantum theoretical calculation.”.*

(German original, Nordheim ([3], p. 512): *“Nach der Quantentheorie kann sich der innere Zustand eines Atoms nur sprunghaft ändern. Es sind nur die diskreten stationären Zustände der Atome möglich. An Stelle der klassischen kontinuierlichen Energieaufnahme haben wir daher eine diskontinuierliche in einzelnen Sprüngen, für die gewisse Übergangswahrscheinlichkeiten bestehen. Es müssen dann zwischen beiden korrespondenzmässige Beziehungen vorhanden sein, d.h. näherungsweise, für grosse Quantenzahlen sogar exakt, Übereinstimmung zwischen der klassischen und quantentheoretischen Rechnung herrschen.”*).

At that time, communication through letters, preprints, travel, and conferences played an important role, and novelties were not always reflected in the publication date. Given the Schrödinger equation (interpreted by Schrödinger [4] as a charge density), the nontrivial part of the statistical interpretation was to show that the statistical properties of scattering experiments were consistent with, and could be derived from, the deterministic Schrödinger equation.

In June 1926, Born ([5], p. 822) found a highly successful statistical interpretation of the, then very new, wave function of Schrödinger, from which he could derive very specific physical conclusions, namely the qualitative features of collision experiments (which were later verified quantitatively). This earned Born the Nobel prize in 1954 *“for his fundamental research in quantum mechanics, especially for his statistical interpretation of the wavefunction”* [6].

Wallner’s Nobel prize presentation speech for the general public commented ([6]) *“Schrödinger had not solved the problem of how it is possible to make statements about the positions and velocities of particles if one knows the wave corresponding to the particle. Born provided the solution to the problem. He found that the waves determine the probability of the measuring results. For this reason, according to Born, quantum mechanics gives only a statistical description.”*. This is a bit inaccurate, as Born found probabilities for state transitions of particles, but not probabilities for positions and velocities of particles—this was achieved a little later by Pauli. This motivated a detailed study of the history of the statistical interpretation of quantum mechanics.

This paper traces the early history of the Born rule 100 years ago, its generalization (essential for today’s quantum optics and quantum information theory) to POVMs 50 years ago, and a modern derivation from an intuitive definition of the notion of a quantum detector. It is based to a large extent on little-known results from my recent books (Neumaier [7] and Neumaier & Westra [8]) and three unpublished preprints (Neumaier [9,10,11]).

### 1.1. The Born Rule Before 1970

The Born rule has many slightly inequivalent formulations, ten different forms of which we state in the following, in modern terminology. Two formulations, the universal form (BR-US) and the discrete form (BR-DS) are taken almost verbatim from Wikipedia [12]. Four other formulations are taken with minor changes from Chapter 14 of Neumaier [7] (but with the scattering form restated in two almost identical formulations with different meaning), and one more from Part II of Neumaier & Westra [8]. Two more general formulations were discovered only after 1970, and are described in Section 1.2. Finally, a tenth form (BR-Q) is presented in Section 1.3, adapted to quantum statistical mechanics and quantum field theory.

**(BR-OSc), Born rule (objective scattering form)**: In a scattering experiment described by the S-matrix *S*,

(1) $$\mathrm{Pr}\left(\psi_{\mathrm{out}}|\psi_{\mathrm{in}}\right):={\left|\psi_{\mathrm{out}}^{*}S\psi_{\mathrm{in}}\right|}^{2}$$

is the conditional probability density that the scattering of particles prepared in the in-state $\psi_{\mathrm{in}}$ results in particles in the out-state $\psi_{\mathrm{out}}$. Here, the in- and out-states are asymptotic eigenstates of the Hamiltonian, labeled by a maximal collection of independent quantum numbers (including the energies, momenta, and angular momenta of the particles involved).

**(BR-OE), Born rule (objective expectation form)**: The value of a quantity corresponding to an operator *X* of a system in the pure state ψ or the mixed state ρ equals on average the quantum expectation value

(2) $$\langle X\rangle :=\psi^{*}X\psi ,$$

or

(3) 〈X〉:=trρX,

respectively. Here, tr denotes the trace of an operator.

**(BR-MSc), Born rule (measured scattering form)**: In a scattering experiment described by the S-matrix *S*, Formula (1) gives the conditional probability density that when particles prepared in the in-state $\psi_{\mathrm{in}}$ are scattered, they are found in the out-state $\psi_{\mathrm{out}}$. Here, the in- and out-states are asymptotic eigenstates of the Hamiltonian, labeled by a maximal collection of independent quantum numbers (including the energies, momenta, and angular momenta of the particles involved).

As part of the Born rule, it is frequently but not always stated that the results of the measurement of a quantity exactly equal one of the eigenvalues.

**(BR-US), Born rule (universal spectral form)**: If a quantity corresponding to a self-adjoint Hermitian operator *X* is measured in a system described by a pure state with normalized wave function ψ, then

(i) the measured result will be one of the eigenvalues λ of *X*, and
(ii) for any open interval Λ of real numbers, the probability of measuring λ∈Λ equals $\psi^{*}P\left(\Lambda \right)\psi$, where P(Λ) is the projection onto the invariant subspace of *X* corresponding to the spectrum in Λ by the spectral theorem.

Since this formulation involves projection operators, measurements satisfying (BR-US) are called **projective measurements**; other names for these are von Neumann measurements and ideal measurements. Not all measurements are of this kind; see Section 1.2 for a more general class of measurements, and Section 3.4 for a fuller discussion of the practical limitations of the class of projective measurements.

**(BR-FS), Born rule (finite spectral form)**: In a projective measurement of a Hermitian, self-adjoint operator *X* (or vector of pairwise commuting operators) with a finite spectrum, the possible values are precisely the finitely many eigenvalues $\lambda_{k}$ of *X* (or joint eigenvalues of the components of *X*), measured with a probability of

(4) $$p_{k}=\mathrm{tr}\;\rho P_{k},$$

where $P_{k}$ is the orthogonal projector to the eigenspace of *X* corresponding to $\lambda_{k}$.

**(BR-DS), Born rule (discrete spectral form)**: Suppose that a quantity corresponding to a self-adjoint Hermitian operator *X* with a discrete spectrum is measured in a system described by a pure state with normalized wave function ψ. Then,

(i) the measured result will be one of the eigenvalues λ of *X*, and
(ii) the probability of measuring a given eigenvalue λ equals $\psi^{*}P\psi$, where *P* is the projection onto the eigenspace of *X* corresponding to λ.

**(BR-ME), Born rule (measured expectation form)**: If a quantity corresponding to a self-adjoint Hermitian operator *X* is measured on a system in the pure state ψ or the mixed state ρ, the results equal, on average, the quantum expectation values (2) or (3), respectively.

### 1.2. The Born Rule After 1970

Nearly 50 years after Born, a theoretical description of more general quantum measurements was introduced in 1970 by Davies & Lewis [13]. A much more readable account was given by Ali & Emch [14] in terms of **positive operator valued measures** (**POVM**s).

These general measurement schemes are based on the concept of a **finite quantum measure** (also called a **finite resolution of unity**), a family of finitely many Hermitian positive semidefinite operators $P_{k}$ on a Hilbert space summing to 1,

$$\sum_{k}P_{k}=1.$$

This straightforward generalization of the concept of a finite probability measure, given by a family of finitely many nonnegative numbers $p_{k}$ (probabilities) summing to 1, is a simplified version of a finite POVM. The following generalization of the finite spectral form (BR-FS) of the Born rule is essentially given in Ali & Emch ([14], (2.14)).

**(BR-POVM), Born rule (POVM form)**: In a general quantum measurement, the possible values are finitely many distinct numbers or vectors $\lambda_{k}$, measured with a probability of (4), where the $P_{k}$ form a quantum measure.

Progressing from the Born rule to so-called positive operator-valued measures (POVMs) is already a big improvement, and commonly used in quantum optics and quantum information theory. Many measurements in quantum optics are POVM measurements [15], i.e., described by a positive operator-valued measure. These follow a different law, of which the Born rule is just a very special case where the POVM is actually projection-valued. Quantum measures are indispensable in quantum information theory (Nielsen & Chuang [16]). They are able to account for things like losses, imperfect measurements, limited detection accuracy, dark detector counts, and the simultaneous measurement of position and momentum.

Quantum measures are also needed to describe quite ordinary experiments without making the traditional textbook idealizations; see, e.g., Busch et al. [17]. For a fairly concise, POVM-based exposition of the foundations of quantum mechanics, see, e.g., Englert [18]. A short history can be found in Brandt [19].

The quantum measure description of a real measurement device cannot simply be postulated to consist of orthogonal projectors. The correct quantum measure must be found by quantum measurement tomography, guided by the theoretical model of the measuring equipment, then ultimately calibrating it using the formula (BR-POVM); see, e.g., D’Ariano et al. [20] and Neumaier [11].

To justify (BR-POVM), the usual practice is to assume the Born rule for projective measurements as a basic premise, with a purely historical justification. Later (if at all), the more general quantum measure (POVM) setting is postulated and justified (e.g., in Fuchs & Peres [21]) in terms of the Born rule for projective measurements, in an extended state space formally constructed using an appropriate ancilla on the basis of Naimark’s theorem (Naimark [22]). This shows consistency with the Born rule, but the ancilla does not always have a meaning in terms of the physical Hilbert space.

In Section 3, following Neumaier & Westra ([8], Part II), we give precise definitions that lead to the correspondence between the statistical expectations and quantum expectations claimed by the following condensed form of the Born rule.

**(BR-C), Born rule (condensed form)**: *The statistical expectation of the measurement results equals the quantum expectation of the measured quantity.*

Both (BR-POVM) and (BR-C) are derived in Section 3.3 from a new, nontechnical measurement principle that is easy to motivate and understand, without any technicalities regarding the spectrum of operators.

### 1.3. Quantum Expectation Values

The quantum expectation values (2) and (3) are purely mathematical definitions, and hence belong to the core of uninterpreted quantum mechanics. Their use in calculations is completely independent of any (assumed or questioned) relations of the quantum mechanical formalism to reality. The latter enters only through some versions of the Born rule.

In quantum statistical mechanics and quantum field theory, one usually makes a theoretical analysis of quantum processes where no actual measurement is made before the process is completed. Since one cannot invoke formulations of the Born rule that make reference to measurement, all probabilities and expectation values that appear in such an analysis, must be considered as formal expressions (2)–(4), with a clear mathematical meaning independent of the interpretation of quantum mechanics in terms of reality, knowledge, or measurement.

Every knowledge-based approach to statistical mechanics needs (BR-C) at the very basis, though this is usually left implicit and only tacitly assumed. Indeed, the only way to know something about the quantum expectation values in an assumed model of a real quantum system is to gather information about the system or its source, and to translate this into information about the state of the system. The latter is usually achieved by means of the **maximum entropy principle**, which (Balian & Balazs [23]) directly equates the statistical expectation value with the quantum expectation value, in order to be able to estimate the state. Note that even Heisenberg’s uncertainty relation is a statement about (theoretical) quantum expectation values that needs (BR-C) to imply anything about (measured) statistical uncertainty.

Since (BR-OSc) and (BR-OE) do not refer to measurement, they are not loaded with the difficulties surrounding the measurement problem of quantum mechanics. However, as we shall discuss in more detail in Section 2.3, they had already been abandoned by 1930, being considered to be mathematically inconsistent. (A referee remarked that Bohmian mechanics restored the consistency of an objective, measurement-free view. But the restoration in Bohmian mechanics was only partial, namely for position, whereas there is still no objective interpretation of the measurement of energy and angular momentum. Thus, (BR-OSc) and (BR-OE) do not hold.)

In particular, without objective interpretation, the quantum probabilities and quantum expectation values are purely theoretical quantities, without any direct relation to reality. The link to reality is exclusively in the subject possessing the knowledge considered.

If one, nevertheless, wants to give the computations in quantum statistical mechanics an objective physical meaning, one must therefore define the objective quantum value of a vector *X* of operators to *be* the quantum expectation value 〈X〉:

**(BR-Q), Born rule (quantum value form)**: The quantum value $\bar{X}$ of a quantity corresponding to an operator *X* of a system in the pure state ψ or the mixed state ρ is defined as the quantum expectation value (2) or (3), respectively. It is approximately measured, with an uncertainty of at least

(5) $$\sigma_{X}:=\sqrt{\langle {\left(X-\bar{X}\right)}^{*}\left(X-\bar{X}\right)\rangle }.$$

Here, $X^{*}$ is the adjoint of *X*.

This form of the Born rule expresses the spirit of Heisenberg’s ([24], p. 181f) statement: *“To every quantum theoretical quantity or matrix, one may associate a number, that gives its ’value’, with a certain probable error; the probable error depends on the coordinate system; for every quantum theoretical quantity there is a coordinate system in which the probable error for this quantity vanishes.”.*

(German original: *“Jeder quantentheoretischen Grösse oder Matrix lässt sich eine Zahl, die ihren ’Wert’ angibt, mit einem bestimmten wahrscheinlichen Fehler zuordnen; der wahrscheinliche Fehler hängt vom Koordinatensystem ab; für jede quantentheoretische Grösse gibt es je ein Koordinatensystem, in dem der wahrscheinliche Fehler für diese Grösse verschwindet.”*) in his 1927 paper on the uncertainty relation.

The quantum value form (BR-Q) of the Born rule is the basis of the thermal interpretation of quantum mechanics, as discussed in my book [7].

In quantum field theory, quantum expectation values of non-Hermitian operators are routinely used; they are needed in the definition of so-called *N*-point functions as quantum expectation values of products of field operators. That 2-point functions are, in principle, observables through linear response theory shows that the operators corresponding to observable quantities need not be Hermitian. Indeed, Dirac’s 1930 quantum mechanics textbook (Dirac ([25], p. 24)), which introduced the name ”observable” for operators, used this terminology for arbitrary linear operators: *“It is convenient to count any operator that can be multiplied into the ψs and ϕs in accordance with the foregoing axioms as an observable.”.*

It is, therefore, interesting to note that all later editions of Dirac’s textbook and *all* later textbooks on quantum mechanics require the restriction to Hermitian operators possessing a real spectral resolution, i.e., in modern terminology, to self-adjoint Hermitian operators. Probably, Dirac became aware of the fact that when *X* is not normal (and, in particular, when *X* is defective, and does hence not even have a spectral resolution), it is impossible to give the quantum value 〈X〉 of *X* a spectral interpretation—which before 1970 was an essential ingredient of all measurement-based forms of the Born rule. Only the more recent formulations (BR-POVM), (BR-C), and (BR-Q) are again free of a spectral restriction.

### 1.4. Discussion

The mathematically consistent relaxation (BR-Q) of (BR-OE) gives up a key assumption tacitly made in classical statistical physics, and imported from there into the spectral forms of the Born rule:

Assumption (D): If the value of *X* is *x*, then the value of f(X) is f(x).

In practice, we only measure a small set of distinguished variables, such as distances, position, energy, momentum, angular momentum, spin, and helicity, and not arbitrary nonlinear expressions in these. Hence, it is quite reasonable to not require (D) for arbitrary nonlinear *f* but only for linear *f*.

Note that giving up (D) is necessary if one wants to account for experiments involving the simultaneous measurement of position and momentum, such as routinely used in modern collision experiments, where particle tracks with fairly well-determined positions and momenta are reconstructed in a time projection chamber. A POVM representation of the latter is given in Neumaier & Westra ([8], Section 6.2.4). Indeed, (D) is also incompatible with (BR-POVM).

The three newest forms of the Born rule, (BR-POVM), (BR-C), and (BR-Q), are mathematically much more elementary, since they do not depend on the notion of the self-adjointness of operators. The POVM form (BR-POVM) of the Born rule is more or less equivalent to the condensed form (BR-C); see Section 3.3. As already stated in Section 1.2, (BR-POVM) is a generalization of the finite spectral form (BR-FS).

The measured expectation form (BR-ME) of the Born rule asserts that measurements result in a random variable whose expectation agrees with the formal quantum expectation. The average in question cannot be taken as a sample average (where only an approximate equal results, with an accuracy depending on the size and independence of the sample) but must be considered as the theoretical expectation value of the random variable. Thus, (BR-ME) implies (BR-C). On the other hand, (BR-C) is more general, since it makes no self-adjointness assumption. For example, (BR-C) applies to complex measurements of the operator A=p+iq, and equivalently to the joint measurement of position *q* and momentum *p*. (By Heisenberg’s uncertainty relation, these cannot be measured jointly with arbitrary accuracy, but projective measurements do not allow them to be jointly measured at all).

Mathematically, the expectation value of a random variable is completely insensitive to the results of a finite number of realizations, just as the limit of a sequence does not change when finitely many sequence entries are changed arbitrarily. Since we can only take finitely many measurements of a system, the measured expectation form (BR-ME) of the Born rule says, strictly speaking, nothing at all about individual measurement results.

If the measurement result $\lambda_{i}$ is an isolated eigenvalue of *A*, the universal form (BR-US) reduces to the discrete form (BR-DS), since one can take Λ to be an open interval only intersecting the spectrum in $\lambda_{i}$, and in this case, $P\left(\Lambda \right)=P_{i}$. The discrete form (BR-DS) clearly implies the finite form (BR-FS) for the case where *A* is a scalar. (BR-FS) takes into account that realistic measurements can only distinguish finitely many measurement results. (BR-FS) also allows for the joint measurement of commuting quantities, but similar extensions could be formulated for the discrete form and the universal form. For projective measurements, (BR-FS) is derived from (BR-C) in Section 3.3.

If, in a projective measurement with one-dimensional projectors $P_{k}=\phi_{k}\phi_{k}^{*}$, the source is pure, described by $\rho =\psi \psi^{*}$ with the normalized state vector ψ∈H, then (4) can be written in the more familiar squared amplitude form

(6) $$p_{k}={\left|\phi_{k}^{*}\psi \right|}^{2}.$$

Together with the relation $\psi =S\psi_{\mathrm{in}}$, which links ψ to the asymptotic in-state via the S-matrix, this shows that the finite spectral form (BR-FS) implies the measured scattering form (BR-MSc).

Using the spectral theorem, it is not difficult to show that the universal form of the Born rule implies the measured expectation form. Conversely, when one assumes condition (D), which effectively forces the measurements to be projective measurements, the measured expectation form (BR-ME) of the Born rule implies the second part (ii) of the universal form (BR-US). It follows that the first part (i) holds with probability 1, but not with certainty, as the universal form requires. This is not just hair splitting. The difference between probability 1 and certainty can be seen by noting that a random number drawn uniformly from [0,1] is irrational with probability 1, while measurements usually produce rational numbers.

von Neumann ([26], p. 255) derived for a quantum expectation value satisfying four (for von Neumann) plausible conditions (conditions A.–D. listed and motivated on pp. 250–252) for the necessity of the formula (3) with a Hermitian density operator ρ (his *U*) of trace 1. This is abstract mathematical reasoning, still within the core of formal (uninterpreted) quantum mechanics, and independent of any relation to measurement. Note that assuming von Neumann’s condition D, which is a restricted form of condition (D) above, forces the measurements to be projective measurements. Thus, he misses the POVM generalization of the Born rule, since the latter violates his condition D.

## 2. The Born Rule 100 Years Ago

### 2.1. The Genesis of the Born Rule

The term ’Born rule’ appeared quite late, apparently first being used in 1934 by Bauer ([27], p. 302) (*“la règle de Born”*; cf. Ziegler [28]). Before that, during the gestation period of finding the right level of generalization and interpretation, the pioneers talked more vaguely about Born’s interpretation of quantum mechanics (or of the wave function). For example, in 1927, Jordan ([29], p. 811) wrote about *“Born’s interpretation of the solution [of the] Schrödinger equation”*.

(German original: *“Born’s Deutung der Lösung [der] Schrödingergleichung”*).

Wessels ([30], p. 187) even goes so far as to claim that *“Neither Born nor most of his contemporaries saw in it anything that even suggests the interpretation that we now associate with his name.”*. Thus, it is interesting to consider the genesis of the Born rule, based on the early papers of the pioneers of quantum mechanics. Mehra & Rechenberg [31] give a detailed history of Born’s statistical interpretation, and Bacciagaluppi [32] expands on the early years 1926–1927. However, they miss a number of subtle points that shed new light on the early developments.

Born originally related his interpretation not to measurement but to objective properties of scattering processes, no matter whether or not these were observed.

The two 1926 papers by Born [5,33] (the first being a summary of the second) introduced the probabilistic interpretation. His 1926 formulation *“determines the probability that the electron cominf from the z-direction is thrown into the direction determined by α,β,γ (with a phase change of δ)”.*

(German original, Born ([33], p. 865f): *“bestimmt die Wahrscheinlichkeit dafür, dass das aus der z-Richtung kommende Elektron in die durch α,β,γ bestimmte Richtung (und mit einer Phasenänderung δ) geworfen wird”*) when rephrased in modern terminology, is a special case of the objective scattering form (BR-OSc) of the Born rule. Note that Born did not have the concept of an S-matrix, first introduced in 1937 by Wheeler [34]. (The S-matrix elements $S_{ik}$ are the inner products $e_{i}^{*}Se_{k}$, where the $e_{i}$ are the standard basis vectors of the matrix *S*; in Born’s case, the stationary states of the particle. The relation with the S-matrix is more explicitly visible in Dirac [35], who computed in 1927 what are today called the S-matrix elements in the so-called Born approximation.) However, the square of the absolute value the S-matrix elements for single-particle scattering is proportional to the *“Ausbeutefunktion”* $\Phi_{n,m}$ mentioned in Born ([5], p. 824), already used (with similar notation) in Nordheim ([3], p. 512).

Bacciagaluppi [32] comments: *“Importantly, the corresponding probabilities are not probabilities for ’finding’ a system in a certain stationary state upon measurement: the atom and the electron (at least when the interaction is completed) are assumed to be in a stationary state.”*. Thus, Born wrote about objective properties of electrons (“being thrown out”) independent of measurement. His statement does not depend on anything being measured, let alone to assigning a precise numerical measurement value!

The 1927 paper by Born [36] extends this rule on p. 173 to probabilities for quantum jumps (”Quantensprung”, p. 172) between energy eigenstates, given by the absolute squares of inner products of the corresponding eigenstates, still using objective rather than measurement-based language: For a system initially in state *n* given by formula (9) in Born’s paper, he says *“The square $|b_{nm}{|}^{2}$ is, according to our basic hypothesis, the probability that after the end of the disturbance, the system is in state m”*.

(German original: *“Das Quadrat $|b_{nm}{|}^{2}$ ist gemäss unserer Grundhypothese die Wahrscheinlichkeit dafür, dass das System sich nach Ablauf der Störung im Zustand m befindet”*). Here, state *n* is the *n*th stationary state (eigenstate with a time-dependent harmonic phase) of the Hamiltonian.

Born derives this rule from two assumptions. The first assumption, made on p. 170 and repeated on p. 171 after (5), is that an atomic system is always in a definite stationary state: *“Thus we shall stick to Bohr’s picture that an atomic system is always only in a stationary state. […] but in general we will know in any moment only that, because of the prior history and the given physical conditions, there is a certain probability that the atom is in the nth state.”.*

(German original: *“Wir werden also an dem Bohrschen Bilde festhalten, dass ein atomares System stets nur in einem stationaren Zustand ist. […] im allgemeinen aber werden wir in einem Augenblick nur wissen, dass auf Grund der Vorgeschichte und der bestehenden physikalischen Bedingungen eine gewisse Wahrscheinlichkeit dafür besteht, dass das Atom im n-ten Zustand ist.”*).

Thus, for the early Born, the quantum numbers of the (proper or improper) stationary states of the system are objective properties of a quantum mechanical system. This assumption indeed works for equilibrium quantum statistical mechanics—where expectations are defined in terms of the partition function and a probability distribution over the stationary states. It also works for nondegenerate quantum scattering theory—where only asymptotic states figure. However, it has problems in the presence of degeneracy, where only the eigenspaces, but not the stationary states themselves, have well-defined quantum numbers. Indeed, Born assumes—on p. 159, in a remark after his (2) and his Footnote 2—that the Hamiltonian has a nondegenerate, discrete spectrum.

Born’s second assumption is his basic hypothesis on p. 171 for probabilities of being (objectively) in a stationary state: *“[…] is a certain probability that the atom is in the nth state. We now claim that as measure for this state probability, one has to choose the quantity $|c_{n}{|}^{2}={\left|\int \psi \left(x,t\right)\psi_{n}^{*}\left(x\right)dx\right|}^{2}$.”*

(German original: *“[…] eine gewisse Wahrscheinlichkeit dafür besteht, dass das Atom im n-ten Zustand ist. Wir behaupten nun, dass als Mass dieser Zustandswahrscheinlichkeit die Grösse $|c_{n}{|}^{2}={\left|\int \psi \left(x,t\right)\psi_{n}^{*}\left(x\right)dx\right|}^{2}$ zu wählen ist.”*). This (now obsolete) interpretation of objective stationary states persisted for some time in the literature; e.g., in the 1929 book by de Broglie [37]. In the introduction, he refers both to an objective position-based and to a measurement-based spectral formulation:

p. 4: *“In the proper domain of the new dynamics, one can usually rely on the principle that the square of the wave amplitude, the intensity, at a particular place and a particular time gives the probability that the particle is at this time at this place. One ckecks easily that this principle is necessary to explain diffraction and interference.”*.

(German original: *“Im eigentlichen Gebiet der neuen Dynamik kann man sich meistens auf das Prinzip verlassen, dass das Quadrat der Wellenamplitude, die Intensität, an einem bestimmten Ort und zu einer bestimmten Zeit die Wahrscheinlichkeit dafür liefert, dass sich das Teilchen zu dieser Zeit an diesem Ort befindet. Man überlegt leicht, dass dieses Prinzip notwendig ist, um Beugung und Interferenz zu erklären.”*).

On p. 6f, after mentioning difficulties of the objective position-based view: *“Finally, there is still a fourth approach, which currently has the most supporters. It was developed by Heisenberg and Bohr. […] According to this view, the wave […] is only a symbolic representation of what we know about the particle. […] Thus the intensity distribution and spectral nature of the wave allws one to give the probability that an experiment performed at time t finds the particle at a particular place, or assigns to it a particular state of motion.”*.

(German original: *“Schliesslich gibt es noch eine vierte Betrachtungsweise, die augenblicklich die meisten Anhänger hat. Sie wurde von Heisenberg und Bohr entwickelt. […] Nach dieser Auffassung ist die Welle […] nur eine symbolisch Darstellung von dem, was wir über das Teilchen wissen. […] so gestatten Intensitätsverteilung und Spektralbeschaffenheit der Welle, die Wahrscheinlichkeit dafür anzugeben, dass ein zur Zeit t ausgeführtes Experiment das Teilchen an einem bestimmten Ort findet oder ihm einen bestimmten Bewegungszustand zuschreibt.”*).

The objective interpretation is needed to be able to argue on p. 70, as follows: *“The density of the ensemble can also be regarded as the probability that a particle, whose motion belongs to the class considered, is at a particular time at a particular point, if its exact position is unknown. […] We had denoted this assumption in the introduction as ‘interference principle’.”*

(German original: *“Die Dichte des Schwarms kann man auch als die Wahrscheinlichkeit dafür betrachten, dass ein Teilchen, dessen Bewegung zur betrachteten Klasse gehört, sich zu einer bestimmten Zeit an einem bestimmten Punkt befindet, wenn seine genaue Lage unbekannt ist. […] Wir hatten diese Annahme in der Einleitung als ‘Interferenzprinzip’ bezeichnet.”*) However, de Broglie mentions at several places the associated difficulties.

In the main text, he mentions on p. 117f, on the one hand, $a^{2}={\left|\psi \left(x\right)\right|}^{2}$ (*“by the interference principle”* of Schrödinger, pp. 3–5), and, on the other hand, $a_{k}^{2}$ (*“by the postulate of Born”*), now in an objective formulation: *“When the wave mechanics was still at the beginning of its development, Max Born already made the proposal to regard every quantity $a_{k}^{2}$ as the relative probability for a particle to have the state of motion belonging to ψ. […] We call this postulate of Born the ’principle of spectral resolution’. Assuming its validity, the determination of the particle through the corresponding wave has a double uncertainty: On the one hand, the position of the particle is undetermined, since by the interference principle, there is a positive probability that one finds the particle in any place of the spatial region occupied by the wave packet, being equal to the resulting intensity $a^{2}$. On the other hand, the state of motion, measured through momentum and energy, is by the principle of spectral resolution also undetermined, since there are several possible states of motion, and the probability for each one is the amplitude square of the corresponding monochromatic component in the spectral resolution of the wave packet.”*.

(German original: *“Als die Wellenmechanik noch am Anfang ihrer Entwicklung stand, hat Max Born schon den Vorschlag gemacht, jede Grösse $a_{k}^{2}$ als die relative Wahrscheinlichkeit dafür aufzufassen, dass das Teilchen den zu ψ gehörigen Bewegungszustand hat. […] Wir nennen dieses Bornsche Postulat das ’Prinzip der spektralen Zerlegung’. Nimmt man seine Gültigkeit an, so hat die Bestimmung des Teilchens durch die zugehörige Welle eine doppelte Unbestimmtheit: einerseits ist die Lage des Teilchens unbestimmt, da es nach dem Interferenzprinzip eine endliche Wahrscheinlichkeit gibt, dass man das Teilchen an irgendeiner Stelle des Raumgebiets findet, das vom Wellenzug ausgefüllt wird, und zwar ist sie gleich der resultierenden Intensität $a^{2}$. Andererseits ist der durch Impuls und Energie gemessene Bewegungszustand nach dem Prinzip der spektralen Zerlegung ebenfalls unbestimmt, weil es mehrere mögliche Bewegungszustände gibt und die Wahrscheinlichkeit für jeden durch das Amplitudenquadrat der zugehörigen monochromatischen Komponente in der spektralen Zerlegung des Wellenzugs ist.”*).

De Broglie, here, uses exactly the same terminology as Born did in his 1927 paper ([36], p. 168), *“The particles are always accompanied by a wave process; these de Broglie–Schrödinger waves depend on the forces and determine through the square of their amplitude the probability that a particle is present in the state of motion corresponding to the wave. In hist last communication, Schrödinger also discussed the amplitude square of the waves and introduces for it the term ‘weight function’, which is already very close to the terminology of statistics.”.*

(German original: *“Die Teilchen sind immer von einem Wellenvorgang begleitet; diese de Broglie–Schrödingerschen Wellen hängen von den Kräften ab und bestimmen durch das Quadrat ihrer Amplitude die Wahrscheinlichkeit dafür, dass ein Teilchen in dem der Welle entsprechenden Bewegungszustand vorhanden ist. In seiner letzten Mitteilung beschäftigt sich auch Schrödinger mit dem Amplitudenquadrat der Wellen und führt dafür den Ausdruck ‘Gewichtsfunktion’ ein, der sich der Terminologie der Statistik schon sehr nähert.”*). Thus, we know that *“Bewegungszustand”* (“state of motion”) stands for the energy–momentum eigenstate of the particle. Indeed, “state of motion” was at that time a technical term, and now no longer in use. The 1898 book by Jordan ([38], p. 1f) defines *“With the word ’force’, one denotes the cause of a change of the state of motion of a body.—As state of motion one has to understand not only a motion of any kind but also the rest—as lack of any motion (velocity = 0, cf. p. 3). […] Every body maintains without change the state of motion that it has at any motion, in direction and speed, as long as no external force affects a change.”.*

(German original: *“Mit dem Worte Kraft bezeichnet man die Ursache einer Änderung des Bewegungszustandes eines Körpers.—Als Bewegungszustand ist nicht nur eine Bewegung irgend welcher Art, sondern auch die Ruhe—als Abwesenheit jeglicher Bewegung (Geschwindigkeit = 0, vgl. S. 3)—aufzufassen. […] Jeder Körper behält den Bewegungszustand, den er in irgend einem Momente hat, nach Richtung und Geschwindigkeit unverändert bei, so lange keine äussere Kraft (ändernd) auf ihn einwirkt.”*). Thus, Bewegungszustand is a state that persists unless changed by an interaction. In 1925, Kramers & Heisenberg ([39], p. 692) used the term in the quantum context for stationary states: *“which refer to two states of motion for which the values of the quantities $J_{1}\cdots J_{s}$ differ by $\tau_{1}h\cdots \tau_{s}h$. […] We represent the states of motion by points in a $J_{1},J_{2}$-plane, the drawing plane of the figure.”.*

(German original: *“die sich auf zwei Bewegungszustände beziehen, für die die Werte der Grössen $J_{1}\cdots J_{s}$ sich um $\tau_{1}h\cdots \tau_{s}h$ unterscheiden. […] die Bewegungszustände seien durch Punkte in einer $J_{1},J_{2}$-Ebene, der Zeichenebene der Figur, dargestellt.”*).

### 2.2. Early Objective, Measurement-Independent Formulations

From the preceding, we may summarize **Born’s statistical interpretation of the wave function** in the version of 25 June 1926 by the following five assertions (expressed in modern terms):

(B1) A state of a quantum system at time *t* is a stationary state, given by a simultaneous eigenstate of *H*, p, and $J^{2}$ (Bacciagaluppi [32] comments this in his footnote 9, as follows: *”This is not in fact stated explicitly but has to be read between the lines.”*. The preceding subsection may, thus, be viewed as giving details on how to read between the lines).

(B2) The corresponding state of motion is given by the joint eigenvalues E=ħω, ħk, and ${\;\hbar }^{2}j\left(j+1\right)$ of the commuting operators *H*, p, and $J^{2}$. In the center of mass frame, p=0. Then, the quantum numbers are determined by *E* and nondegeneracy. (Degenerate cases are excluded by assumption.)

(B3) Every quantum system is at each time *t* in a unique (stationary) state, changing at random times by making a transition (a **quantum jump**). This is deemed obvious from spectroscopic experience.

(B4) Energy and momentum are exactly conserved.

(B5) Associated with each quantum system is a guiding (de Broglie–Schrödinger) wave that determines through its spectral decomposition the probability of being in a particular state.

Note the objective formulation of all statements, without any reference to measurement.

Born’s interpretation met with strong resistance from Schrödinger, who allegedly said—as Heisenberg ([40], p. 6) recalled much later—at a conference in Kopenhagen in September 1926, *“But should this damned quantum jumping persist, then I regret having even worked on this subject.”.*

(German original: *”Wenn es doch bei dieser verdammten Quantenspringerei bleiben soll, dann bedauere ich, dass ich mich überhaupt mit diesem Gegenstand beschäftigt habe.”*). An exchange of letters between him and Born displayed the two extreme poles between which the quantum community had to find a consensus in the subsequent years.

Letter of Schrödinger to Born (2 November 1926) (von Meyenn ([41], p. 329)): *“I just skimmed (hence not yet really read) your last, just received, paper on the adiabatic theorem. […] But I still have the impression that you, and others who essentially share your view, are too deep under the spell of those concepts (such as stationary states, quantum jumps, etc.) that in the last twelve years established their right to be present in our thought, to give full justice to the attempt to escape again from this thought pattern. As an example I mention, e.g., that to the critique of the assumption that in the atom several eigen oscillations are simultaneously excited, you ask the question, does it make sense to say the atom is simultaneously in several eigen oscillations? Another remark seems to be to you a long way off: Always and everywhere else, where we have to do with oscillating systems, these generally oscillate not in the form of an eigen oscillation but in a superposition of these.”.*

(German original: *“Ich habe eben Ihre letzte, ebenerhaltene Arbeit über den Adiabatensatz überflogen (also noch nicht wirklich gelesen). […] Ich habe aber doch den Eindruck, dass Sie und andere, die im Wesentlichen Ihre Ansicht teilen, zu tief im Banne derjenigen Begriffe stehen (wie stationäre Zustände, Quantensprünge usw.), die sich in den letzten zwölf Jahren Bürgerrecht in unserem Denken erworben haben, um dem Versuch, aus diesem Denkschema wieder herauszukommen, volle Gerechtigkeit widerfahren zu lassen. Als Beispiel führe ich z. B. an, dass Sie zur Kritik der Annahme: im Atom seien mehrere Eigenschwingungen gleichzeitig erregt, die Frage stellen: hat es Sinn, zu sagen, das Atom befinde sich gleichzeitig in mehreren stationären Zuständen? Ganz ferne scheint Ihnen die andere naheliegende Bemerkung zu liegen: immer und überall sonst, wo wir mit schwingungsfähigen Systemen zu tun haben, schwingen dieselben im Allgemeinen nicht in der Form einer Eigenschwingung, sondern mit einem Gemisch derselben.”*).

Letter of Born to Schrödinger (6 November 1926): (von Meyenn ([41], p. 333f)): *“As most direct evidence of the stationary states we have the electron collisions, and here I think primarily of collisions of the second kind. We may take, I think, as fact that an electron colliding with an atom in some particular state always gets only one increase in energy. Suppose now that in an atom several eigen oscillations can be simultaneously excited, how should one understand this? What is the use of your conter argument that the electron could itself be a ‘wave group’? For it is the atomic waves that correspond to discrete steps. Of course it is possible to say: The aton that oscillates in several frequencies responds in a special way, such that it transmits to the electron only the energy corresponding to one frequency. But this is only an excuse. It seems to me that Bohr’s way of speaking is the natural description and summary of a large body of facts; therefore it is the task of every finer theory to justify this way of speaking.”.*

(German original: *“Als direkteste Evidenz der stationären Zustände haben wir die Elektronenstösse, und hier denke ich vor allem an die Stösse zweiter Art. Wir können wohl als Tatsache nehmen, dass ein Elektron beim Zusammenstoss mit einem Atom, das in irgend einem Zustande ist, immer nur einen Energiezuwachs bekommt. Wenn nun in einem Atom mehrere Eigenschwingungen gleichzeitig angeregt sein könnten, wie sollte man das verstehen? Was nützt da Ihr Gegenargument, dass das Elektron selber eine ’Wellengruppe’ sein könne? Es handelt sich doch um die Atomwellen, die diskreten Stufen entsprechen. Natürlich ist es möglich zu sagen: das in mehreren Frequenzen schwingende Atom reagiert eben in besonderer Weise so, dass es nur die einer Frequenz entsprechende Energie auf das Elektron überträgt. Aber das ist doch nur eine Ausrede. Mir scheint, dass die Bohrsche Redeweise die natürliche Beschreibung und Zusammenfassung eines grossen Tatsachenbereichs ist; darum ist es Aufgabe jeder feineren Theorie, diese Redeweise zu rechtfertigen.”*).

In general, however, the statistical interpretation was quickly taken up by the quantum community. For example, on 3 November 1926, Fowler [42] submitted a paper stating Born’s interpretation: “The state of the assembly is then specified of systems in the different quantum states, $n_{1},n_{2},\ldots ,n_{u},\ldots$.” (p. 434). Later in the same paper, he speculates (on p. 447) about the position probability density: “We venture in conclusion some speculations on the subject of the statistical distribution of the particles in space. It is of some importance to remember that in the new statistics space distribution laws as such have almost ceased to exist, and this is probably in accord with the requirements of the new mechanics. The primary distribution laws are concerned only with the distribution over the characteristics—that is, the energy. At the same time, there must be some means of deriving the average number of molecules ‘present’ in a given volume element.”.

At that time, Pauli had already obtained the position probability density interpretation, but only in a letter to Heisenberg:

Letter of Pauli to Heisenberg, 19 October 1926 (Pauli ([43], p. 347)): *“Now it is thus: all diagonal elements of the matrices (at least of functions of p alone or of q alone) one can interpret kinematically already now. For one can first ask for the probability that in a particular stationary state, the coordinates $q_{k}$ of the particles (k=1,…,f) lie between $q_{k}$ und $q_{k}+dq_{k}$. The answer is $|\psi \left(q_{1},\ldots ,q_{f}\right){|}^{2}dq_{1}\ldots dq_{f}$, if ψ is Schrödinger’s eigenfunction.”.*

(German original: *“Nun ist es so: alle Diagonalelemente der Matrizen (wenigstens von Funktionen der p allein oder der q allein) kann man überhaupt schon jetzt kinematisch deuten. Denn man kann ja zunächst nach der Wahrscheinlichkeit fragen, dass in einem bestimmten stationären Zustand des Systems die Koordinaten $q_{k}$ seiner Teilchen (k=1,…,f) zwischen $q_{k}$ und $q_{k}+dq_{k}$ liegen. Die Antwort hierauf ist $|\psi \left(q_{1},\ldots ,q_{f}\right){|}^{2}dq_{1}\ldots dq_{f}$, wenn ψ die Schrödingersche Eigenfunktion ist.”*).

The position probability density interpretation was published in the next year in a footnote of Pauli ([44], p. 83, Footnote 1): *“We want to interpret this […] function in the spirit of the ghost field view of Born given in his book [5,33] as follows: $|\psi \left(q_{1}\ldots q_{f}\right){|}^{2}dq_{1}\ldots dq_{f}$ is the probability that in the respective quantum state of the system these coordinates lie simultaneously in the respective volume element $dq_{1}\ldots dq_{f}$ of position space.”.*

(German original: *“Wir wollen diese […] Funktion im Sinne der von Born in seiner Stossmechanik [5,33] vertretenen Auffassung des “Gespensterfeldes” folgendermassen deuten: Es ist $|\psi \left(q_{1}\ldots q_{f}\right){|}^{2}dq_{1}\ldots dq_{f}$ die Wahrscheinlichkeit dafür, dass im betreffenden Quantenzustand des Systems diese Koordinaten sich zugleich im betreffenden Volumenelement $dq_{1}\ldots dq_{f}$ des Lageraums befinden.”*). Apart from its objective formulation (no reference to measurement), this is a special case of the universal formulation (BR-US) of the Born rule.

The 1927 paper by von Neumann ([45], p. 45) generalized Pauli’s statement to arbitrary self-adjoint operators, again stated as an objective (i.e., measurement independent) interpretation. For discrete energy spectra and their energy levels, we still read p. 48: *“unquantized states are impossible”*.

(German original: *“nicht gequantelte Zustände sind unmöglich”*). Thus he follows Born’s view that all physically possible states are stationary.

Like Born, both Jordan and von Neumann talk about objective properties of the system independent of measurement. But unlike Born, who ties these properties to the stationary state representation in which momentum and energy act diagonally, Pauli ties them to the position representation, where position acts diagonally, and von Neumann allows this for arbitrary systems of commuting self-adjoint operators.

From either of Born’s or Pauli’s statements, one can easily obtain the basis-independent objective expectation form (BR-OE) of the Born rule, either for functions *A* of stationary state labels, or for functions *A* of position.

The first published statement of (BR-OE) seems to be the 1927 paper by Landau [46]. His formulas (4a) and (5), corresponding in modern notation to 〈A〉:=trρA in the mixed case and $\langle A\rangle :=\psi^{*}A\psi$ in the pure case, $\rho =\psi \psi^{*}$, are interpreted in his Footnote 2 as probability mean (German original: “*Wahrscheinlichkeitsmittelwert*”). Though he mentions that this rule is already known, earlier formulas in print for the mean always assumed a representation in which the operator is already diagonal. Again, there is no reference to measurement.

### 2.3. Paradoxes and Measurement Context in Born’s Rule

Already in 1926, Pauli ([43], p. 347) noticed the paradox that his objective position probability and Born’s objective momentum probability interpretation following from a spectral interpretation of the objective expectation form (BR-OE) of the Born rule were mutually incompatible. He expressed this in the following words: *“One can view the worlds with the p-eye and one can view it with the q-eye, but if one wants to open both eyes at the same time, one gets crazy”*; German original: *“Man kann die Welt mit dem p-Auge und man kann sie mit dem q-Auge ansehen, aber wenn man beide Augen zugleich aufmachen will, dann wird man irre”.*

The 1927 paper by Jordan ([29], p. 811) cites Pauli [44] and extends Born’s second assumption further to an objective, measurement-independent probability interpretation of inner products (probability amplitudes) of eigenstates of two arbitrary operators, in place of position and momentum, without being aware of the conceptual problem this objective view poses when applied to non-commuting operators.

In 1927, Weyl [47], still using objective language (p. 9: “hat … die Grösse”) and no reference to measurement, pointed out on p.2f that, due to non-commutativity and the resulting complementarity, Jordan’s objectively interpreted formalism is mathematically defective and cannot extend to general operators. The reason is (though Weyl does not say this explicitly) that there is no joint probability density for non-commuting operators, and hence not for position and momentum. In particular, Born’s stationary state probability interpretation and Pauli’s position probability density interpretation cannot both claim objective status.

Still in the same year, von Neumann [26] also noted (on p. 248) the problems resulting from non-commuting quantities that cannot be observed simultaneously, However, unlike Weyl, von Neumann was motivated by a consideration on p. 247 of the measurement of values in an ensemble of systems, taking the expectation to be the ensemble mean of the measured values. Specialized to a uniform (“einheitlich”) ensemble of systems in the same completely known (pure) state ψ of norm state, he then finds on p. 258 that $\rho =\psi \psi^{*}$ with some normalized state vector ψ, giving $\langle X\rangle :=\psi^{*}X\psi$. Note that a logical paradox can only be deduced from (BR-OE) and non-commuting quantities when one assumes von Neumann’s condition D, which forces the measurements to be projective measurements and, therefore, allows one to construct probability densities.

In the present terminology, von Neumann’s interpretation of quantum expectation values is the measured expectation form (BR-ME) of the Born rule. This move eliminated the mathematical paradox noted by Pauli and Weyl, by relating the notion of the value of an observable (being in the *n*th state, or having position *q*) directly to measurement. Only the non-mathematical (hence less dangerous) paradox, that position and momentum (or energy) are not measurable simultaneously to arbitrary precision, was left—a conundrum that had already been given (earlier that year) a precise mathematical form in the uncertainty relation of Heisenberg [24].

Later discussions on the statistical interpretation gradually switched from the objective versions of the Born rule to the measurement-based versions. As we have seen, de Broglie still maintained both variants in 1929. This transition process was apparently completed in 1930 with the appearance of the very influential book by Dirac [25]. A review of the book, by Lennard-Jones [48], stated *“The book contains Dr. Dirac’s philosophy of the relation of theoretical and experimental physics. He believes that the main object of theory is to determine the possible results of an experiment, and to determine the probability that any one of these results will actually occur under given conditions. He regards it as quite unnecessary that any satisfying description of the whole course of the phenomena should be given. A mathematical machine is set up, and without asserting or believing that it is the same as Nature’s machine, we put in data at one end and take out the results at the other. As long as these results tally with those of Nature, (with the same data or initial conditions) we regard the machine as a satisfying theory. But so soon as a result is discovered not reproduced by the machine, we proceed to modify the machine until it produces the new result as well.*

*One might have hoped that the object of the theoretical physics was rather more ambitious than Dirac is willing to allow, and that the steady march forward of physics was taking us further and further forward to a knowledge of the nature of things. But the theoretical physicist, it would seem, must for ever abandon any hope of providing a satisfying description of the whole course of phenomena.”* This is the view that still dominates the philosophy of quantum mechanics today.

### 2.4. Knowledge

Von Neumann’s change of perspective introduced the prominent reference to “knowledge” (von Neumann ([26], p. 247)). It would be interesting to have a thorough historical analysis of the meaning and use of the term knowledge in quantum mechanics. In those days, the term was not understood in a subjective way, but as the objective (through thought experiments theoretically accessible) knowledge of what is real and in principle observable about the system, whether observed or not.

However, the usage of the term “knowledge” created a tension that has never disappeared, and that was verbalized most prominently by Einstein throughout his life. In his obituary on Einstein, Pauli [49] wrote, in 1958 (published in 1959), *“[…] even when I could not agree with Einstein’s views. ’Physics is after all the description of reality’ he said to me, continuing, with a sarcastic glance in my direction ‘or should I perhaps say physics is the description of what one merely imagines?’ This question clearly shows Einstein’s concern that the objective character of physics might be lost through a theory of the type of quantum mechanics, in that as a consequence of its wider conception of the objectivity of an explanation of nature the difference between physical reality and dream or hallucination might become blurred. The objectivity of physics is however fully ensured in quantum mechanics in the following sense. Although in principle, according to the theory, it is in general only the statistics of series of experiments that is determined by laws, the observer is unable, even in the unpredictable single case, to influence the result of his observation—as for example the response of a counter at a particular instant of time. Further, personal qualities of the observer do not come into the theory in any way—the observation can be made by objective registering devices, the results of which are objectively available for anyone’s inspection.”.*

(German original: *“[…] auch wenn ich Einsteins Ansichten nicht zustimmen konnte. ’Physik ist doch die Beschreibung des Wirklichen’, sagte er zu mir und fuhr mit einem sarkastischen Blick auf mich fort: ‘oder soll ich vielleicht sagen, Physik ist die Beschreibung dessen, was man sich bloss einbildet?’ Diese Frage zeigt deutlich Einsteins Besorgnis, dass durch eine Theorie vom Typus der Quantenmechanik der objektive Charakter der Physik verloren gehen könnte, indem durch deren weitere Fassung der Objektivität einer Naturerklärung der Unterschied der physikalischen Wirklichkeit von Traum oder Halluzination veschwommen werden könnte. Die Objektivität der Physik ist in der Quantenmechanik jedoch im folgenden Sinn voll gewahrt. Obwohl nach der Theorie im Prinzip im allgemeinen nur die Statistik von Versuchsreihen gesetzmässig bestimmt ist, kann der Beobachter auch im nicht voraussagbaren Einzelfall das Resultat seiner Beobachtung—wie zum Beispiel das Ansprechen eines Zählers in einem bestimmten Zeitmoment—nicht beeinflussen. Auch gehen persönliche Eigenschaften des Beobachters in keiner Weise in die Theorie ein, vielmehr kann die Beobachtung durch objektive Registrierapparate erfolgen, deren Resultate allen zur Einsicht objektiv vorliegen.”*).

Subjective knowledge (that Einstein hinted at with his question *“or should I perhaps say physics is the description of what one merely imagines?”*) had seriously entered physics only one year earlier, in 1957, with the “subjective statistical mechanics” of Jaynes [50], thus aggravating the tension.

It is easy to see that the amount of knowledge needed to correctly apply the maximum entropy principle must be an intrinsic property of the system modeled. For example, take as a system a turbulent fluid. If one knows only the total energy and the volume, and applies the maximum entropy principle, one gets a Helmholtz ensemble describing a fluid in equilibrium, not the turbulent fluid. Thus, in spite of having used valid knowledge, the maximum entropy principle gives completely wrong results.

To correctly describe a fluid in second quantization with the maximum entropy principle, it is necessary and sufficient that we apply it to an (approximate) knowledge of (at least) the quantum expectation values of the field operators for currents and densities of the fluid at all points in the support of the fluid.

But for a turbulent fluid, one cannot obtain this (very detailed and very quickly changing) knowledge from observation, and hence cannot know it in any meaningful sense! Instead, in all quantum derivations of fluid mechanical equations, one simply assumes that this knowledge (or rather the corresponding maximum entropy state) exists, and then draws consequences through statistical mechanics.

Thus, the knowledge required to fix the state in nonequilibrium statistical mechanics is assumed knowledge, to be checked by its consequences. This makes sense only if the state (and hence any quantum expectation value) assumed in the model is an intrinsic property of the fluid, at least to the extent that the model predictions match observation.

Thus, knowledge is encoded into a particular description, an objective property of the description used to model the system. It is what the particular model claims to know, independently of who “knows” or “uses” it. A change in knowledge is therefore just a change in the details of the model used to describe a particular system. In particular, the situation regarding knowledge is precisely the same as in classical mechanics, where the models also depend on this kind of knowledge (or prejudice, if the model is chosen based on insufficient knowledge).

Therefore, as Pauli had emphasized, nothing personal is implied in the term “knowledge”. There is no more subjectivity in quantum statistical mechanics than in classical engineering physics: The only (and very restrictive) freedom a subject striving for maximal predictivity has is to choose a model correctly describing the system under study. Once the model has been chosen, it determines the quality of the predictions, and, by comparison with corresponding observations, the quality of the knowledge built into the model. If the quality of this knowledge is insufficient, the model is objectively wrong, and was so even before the observations where made.

### 2.5. Collapse (State Reduction)

Tied to the measurement formulations of the Born rule is a controversial **collapse** (or **state reduction**) postulate about the state after a measurement. State reduction was introduced informally in Heisenberg ([24], p. 186) (*“Every determination of position therefore reduces the wave packet again to its original size”*; German original: *“Jede Ortsbestimmung reduziert also das Wellenpaket wieder auf seine ursprüngliehe Grösse”*) and formally established by the authority of Dirac in several editions of his book:

*“The state of the system after the observation must be an eigenstate of [the observable] α, since the result of a measurement of α for this state must be a certainty.”* (Dirac ([25], p. 49), first edition, 1930)

*“Thus after the first measurement has been made, the system is in an eigenstate of the dynamical variable ξ, the eigenvalue it belongs to being equal to the result of the first measurement. This conclusion must still hold if the second measurement is not actually made. In this way we see that a measurement always causes the system to jump into an eigenstate of the dynamical variable that is being measured, the eigenvalue this eigenstate belongs to being equal to the result of the measurement.”* (Dirac ([51], p. 36), third edition, 1936)

Dirac’s statement was discredited (rightly, but without much success) in 1958 by another authority, Landau & Lifshitz ([52], Section 7), who remarked that the state after the measurement is, in general, not an eigenstate. This is apparent in the modern notion of a quantum process (see, e.g., Chuang & Nielsen [53] or Mohseni et al. [54]), which gives the correct post-measurement state for arbitrary POVM measurements.

In 2007, Schlosshauer [55] still took the collapse (*“jump into an eigenstate”*) to be part of what he called the “standard interpretation” of quantum mechanics. But he did not count it as part of the Born rule (p. 35).

It would be interesting to have a more thorough history of the collapse postulate and the extent of its validity.

Closely related to the collapse postulate is the so-called **eigenvalue–eigenstate link** (Fine [56]), thoroughly discussed by Gilton ([57], which asserts that *“if a system has a definite value for X, then the system is in an eigenstate of X.”.*

## 3. The Born Rule Today

The most detailed, universal form of the Born rule is a very complex statement requiring considerable mathematical maturity to be fully understood. Schlosshauer & Fine [58] mention as desirable that a derivation of the Born rule gives *“physical insight into the emergence of quantum probabilities and the Born rule”*. It is indeed strange that such a complicated rule should be part of the basis of quantum theory.

For this and other reasons, there have been a host of attempts to derive the Born rule (in some measured form) from more natural assumptions; see Allahverdyan et al. ([59], Chapter 2) for a thorough discussion of the state of the art until 2013, and Vaidman [60] (2020) for a more recent survey.

All attempts to derive the Born rule are doomed to failure if they do not define, in terms of the formal apparatus of quantum mechanics, the meaning of measurement, a concept appearing in the Born rule but not in the remaining basic rules of quantum mechanics. But measurement is usually introduced tacitly by invoking a seemingly self-evident consequence of the Born rule. For example, use of the maximum entropy principle in a derivation implies circularity, since by the discussion in Section 1.2, its application silently invokes (BR-C).

Another frequent mistake is to derive a probability distribution matching that of Born’s rule, without any actual measurement (with definite results) being involved. Only calculations performed within the theoretical machinery are performed, and only handwaving is used to claim that these calculations have anything to do with measurement. This was the case in the early, decoherence-based ‘derivations’ now known (Schlosshauer [55]) to lack an essential ingredient, the solution of the “unique outcome problem”.

Therefore, all derivations (known to me) found in the literature are either circular or unrelated to measurement, and hence of questionable value.

As examples, we consider three proposed derivations that have appeared in the last few years.

Schonfeld [61] (2021) assumes, in his elementary (but somewhat heuristic) ‘derivation’ of Born’s rule on p.4, the seemingly self-evident fact that the thermal randomness of subcritical droplet formation *“exists independent of any measurement axioms, i.e., independent of whether anyone observes it.”.* While his description gives some insight into the origin of the Born rule, the problem of deriving how random nucleation (rather than a mixture of all possible nucleations) arises from a quantum statistical model (cf. Section 3.5) is not even touched. The traditional statistical account of nucleation by Langer [62] is purely classical. A quantum derivation of nucleation was attempted, e.g., by Lombardo et al. [63]. But (like many others) they assumed without discussion that (under reasonable conditions) the *“Wigner function can then be interpreted as a classical probability distribution for coordinates and momenta”*, an assertion that can be maintained as referring to a statistical distribution only by invoking the condensed version (BR-C) of the Born rule.

Chanda & Bhattacharyya [64] (2021) claim on p. 2 the emergence of Born’s rule in *“a dynamical model without an explicit invocation of an apparatus”*. Thus, they fall into the second trap mentioned above, where nothing ever is measured in their analysis. (In addition, they assume in their derivation, not the unitary dynamics of a closed system, but the dissipative Lindblad dynamics, which would have to be derived without using Born’s rule.).

Aharonov & Shushi [65] (2024) explicitly assume in their derivation—in the line after (1)—that measurement outcomes are eigenvalues, which is part of all spectral forms of the Born rule, and therefore should be proved in a complete derivation. In the line after (9), an observed value comes out of the blue by simple handwaving. (In addition, they explicitly assume a postulate, detailed after (12), which makes claims about unobservable properties of a particle between measurements—without even spelling out what a property should be. Thus, instead of Born’s rule with its clear operational meaning, they have to postulate an obscure assumption unlikely to be acceptable to many physicists.).

### 3.1. The Detector Response Principle

In order not to follow into the trap of circularity, it is necessary to make the place where measurement enters explicit, and to define the properties a detector must have, in order that its observations count as measurements. Therefore, we begin with a precise conceptual framework, within which some versions of the Born rule can be derived.

Following Neumaier & Westra ([8], Part II), we give precise definitions for quantum expectations, statistical expectations, and quantum measurement devices, and derive from these the formulations (BR-POVM) and (BR-C) of the Born rule.

We model a **quantum system** at the formal level using as **state space** H a Hermitian vector space (i.e., a dense subspace of a Hilbert space), and write E:=LinH for the space of everywhere-defined linear operators on H. For single-particle states, H is the Schwartz space of smooth functions of position whose Fourier transform is also smooth. Then, E is an operator algebra which contains the components of position *q* and momentum *p*, and hence contains all normally ordered polynomials in *q* and *p*.

We characterize a **quantum source** by a positive semidefinite Hermitian **density operator** (or **density matrix** if $H=C^{n}$) ρ∈LinH. The **state** of the source is the positive linear mapping 〈·〉 that assigns to each X∈LinH its quantum value

(7) 〈X〉:=trρX.

As is customary, we also refer to ρ as the **state**, since states and density operators are in a 1-to-1 correspondence. More generally, given a fixed state 〈·〉, the **quantum value** of a vector $X\in E^{m}$ with operator components $X_{j}\in E$ is the vector

$$\bar{X}=\langle X\rangle \in C^{m}$$

with components ${\bar{X}}_{j}=\langle X_{j}\rangle$. Any property of the source relevant for quantum detection can be expressed as a function of quantum values. We call the number

I:=〈1〉=trρ

the **intensity** of the source. The intensity is nonnegative, since ρ is positive semidefinite. ρ=0 defines the **empty state**; it is the only state with zero intensity. Note the slight difference compared to conventional density operators, where the trace is instead fixed as one (In contrast to our convention of normalizing the trace of ρ to the intensity, it is customary in the literature to normalize the intensity to be 1. This can be achieved by dividing ρ and all quantum values by the intensity. A disadvantage of this normalization is that ρ loses one degree of freedom and the intensity of the source is no longer represented by the state).

A source and its state are called **pure** if the density operator has rank 1, and hence is given by $\rho =\psi \psi^{*}$ for some **state vector** ψ; if the source or state is not pure, it is called **mixed**. In a pure state, the quantum value takes the form

$$\langle X\rangle =\mathrm{tr}\;\psi \psi^{*}X=\psi^{*}X\psi .$$

Since experimentally realizable detectors always produce only a finite number of possible results, we make the following definition. A **quantum measurement device** (in the following, just called a **measurement device**) is characterized by a collection of **detection elements** labeled by labels *k* from a finite set *K* satisfying the **detector response principle**, given by the following postulate.

**(DRP)**: *A detection element k responds to an incident stationary source with density operator ρ with a nonnegative mean rate $p_{k}$ depending linearly on ρ. The mean rates sum to the intensity of the source. Each $p_{k}$ is positive for at least one density operator ρ.*

A slightly less precise version had already been given in my unpublished 2019 manuscript ([9], p. 8). Note that, in order that a measured mean rate has a sensible operational meaning, the source must be reasonably stationary, at least during the time in which measurements are taken.

Unlike the Born rule, the DRP is not a postulate added to the formal core of quantum mechanics. Instead, it defines what it means for a physical (quantum) object to be a quantum detector. The DRP gives a simply property that decides whether or not a piece of matter may be regarded as a quantum detector, and whose validity can be experimentally tested in concrete cases. Thus, it is fully compatible with the whole mathematical apparatus of quantum mechanics (including unitary evolution), without the need to ‘shut up and calculate’.

The abundant existence of quantum measurement devices satisfying the DRP is an extremely well-established empirical fact. The immediate physical significance of the DRP makes it an excellent starting point for the statistical interpretation of quantum physics; in particular, it leads to a simple, transparent derivation of the Born rule.

The DRP is valid whenever the discrete form (BR-DS) of the Born rule can be expected to hold, and allows us to give (in Section 3.3) a straightforward derivation of the condensed form (BR-C) of the Born rule.

As shown in Neumaier & Westra ([8], Part II), the DRP leads naturally to all basic concepts and properties of modern quantum mechanics. In particular, it gives a precise operational meaning to quantum states, quantum detectors, quantum processes, and quantum instruments. This gives a perspective on the foundations of quantum mechanics that is quite different from the well-trodden path followed by most quantum mechanics textbooks.

### 3.2. The Statistical Interpretation of Quantum Mechanics

The key result for the theory of quantum measurements is the following **detector response theorem**, proved in ([8], Section 4.1.2).

**Theorem** **1.** *For every measurement device, there is a unique discrete quantum measure $P_{k}$ (k∈K) whose quantum values $\langle P_{k}\rangle$ determine, for every source with density operator ρ, the mean rates*

(8) $$p_{k}=\langle P_{k}\rangle =\mathrm{tr}\;\rho P_{k}\;\;\;for\;k\in K.$$

That the $P_{k}$ form a **discrete quantum measure** means that they are Hermitian, positive semidefinite, and sum up to the identity operator 1. This is the natural quantum generalization of a discrete probability measure, a collection of nonnegative numbers that sum up to 1.

The detector response theorem characterizes the response of a quantum measurement device in terms of a quantum measure. A quantum measurement device produces, in the low intensity case, a stochastic sequence of **detection events**, but makes no direct claims about values being measured. It just says which one of the detection elements making up the measurement device responded at which time.

To go from detection events to measured numbers, one needs to provide, in addition, a **scale** that assigns to each detection element *k* a real or complex number (or vector) $x_{k}$. We call the combination of a measurement device with a scale a **quantum detector**. We say that the detector **measures** the **quantity**

(9) $$X:=\sum_{k\in K}x_{k}P_{k}.$$

The same quantum measurement device, equipped with different scales, measures different quantities. When the density operator is normalized to intensity one, the response rates $p_{k}$ form a discrete probability measure. In this case, we refer to a response rate as response probability and to the quantum value of *X* as the quantum expectation of *X*.

These values can be arbitrary numbers or vectors $x_{k}$ (or even more complex mathematical entities from a vector space)—whatever has been written on the scale a pointer points to, or whatever has been programmed to be written by an automatic digital recording device.

A quantum detector may be considered as a technically precise version of the informal notion of an **observer** that figures prominently in the foundations of quantum mechanics. It removes from the latter term all anthropomorphic connotations.

### 3.3. Derivation of the Born Rule

If the intensity is normalized to one, then $\sum_{k}p_{k}=1$, so that the normalized mean rate $p_{k}$ can be interpreted as the **response probability** of detector element *k* given a certain response, or as the **detection probability** for the *k*th detection event. This gives an intuitive meaning for the $p_{k}$ in the case of low intensity measurements. Formula (8), derived here from very simple first principles, becomes (4), but with $P_{k}$ not restricted to projections. Thus, we have proved the POVM form (BR-POVM) of the Born rule. It gives the theoretical quantum values $\langle P_{k}\rangle$ a statistical interpretation as response probabilities $p_{k}$ of a quantum measurement device. Note that all probabilities are classical probabilities in the sense of Kolmogorov [66], as used everywhere in statistics, and can be approximated by relative frequencies with an accuracy given by the law of large numbers.

The results of a detector in a sequence of repeated events define a random variable or random vector $x_{k}$ that allows us to define the **statistical expectation**

(10) $$E\left(f\left(x_{k}\right)\right):=\sum_{k\in K}p_{k}f\left(x_{k}\right)$$

of any function $f(x_{k})$. As always in statistics, this statistical expectation is operationally approximated by finite sample means of f(x), where *x* ranges over a sequence of actually measured values. However, the exact statistical expectation is an abstraction of this; it works with a nonoperational probabilistic limit of infinitely many measured values, so that the replacement of relative frequencies in a sample by probabilities is justified.

From (8) and (9), we find the formula

(11) $$E\left(x_{k}\right)=\mathrm{tr}\;\rho X=\langle X\rangle$$

for the statistical expectation of the measurement results $x_{k}$ obtained from a source with density operator ρ. Comparing with (7), we see that the statistical expectation of the measurement results coincides with the theoretical quantum value of *X* evaluated in the state ρ of the source. This is the measured expectation form (BR-ME) of the Born rule. It gives the purely theoretical notion of a quantum value an operational statistical interpretation in terms of expectations of the measurement results of a quantum detector. If we call the quantum value 〈X〉 the **quantum expectation** of *X*, we find the Born rule (11) in the condensed form (BR-C).

It is shown in ([8], Part II) that the DRP, together with techniques from quantum tomography, allow one to reconstruct from the DRP the complete edifice of quantum mechanics, including its dynamical and spectral consequences. In particular, the DRP is precisely what is needed to obtain concepts and results from quantum information theory.

According to our discussion, the possible values obtained when measuring a particular quantity *X* depend on the decomposition (9) used to construct the scale. That this decomposition is ambiguous follows from the fact that the scale is not determined by the quantity *X* measured. Since the scale determines the measurement results, this means that one can, with equal right, ascribe different results to the measurement of the same quantity *X*. Thus, different detectors measuring the same quantity *X* may have different sets of possible measurement results. In other words, the same quantity *X* can be measured by detectors with different mathematical characteristics, and in particular with different measurement results, which need not have anything to do with the eigenvalues of *X*. In the terminology of Section 2.5, this means that the eigenstate–eigenvalue link is broken.

This is in full agreement with the standard recipes for drawing inferences from inaccurate measurement results. Just like in classical mechanics, they are emergent imperfections due to the experimental conditions in which the measurements are made. The situation is precisely the same as in classical metrology, where observable quantities always have a true value determined by the theoretical description, and all randomness in measurements is assumed to be due to measurement noise.

We now connect our developments to the traditional spectral view of quantum measurements. We call a discrete quantum measure **projective** if the $P_{k}$ satisfy the **orthogonality relations**

(12) $$P_{j}P_{k}=\delta_{jk}P_{k}\;\;\;for\;j,k\in K.$$

We say that a detector measuring *X* performs a **projective measurement** of *X* if its quantum measure is projective.

In practice, the orthogonality relations (12) can often be implemented only approximately (due to problems with efficiency, losses, inaccurate preparation of directions, etc.). Thus, projective measurements are unstable under imperfections in the detector. Therefore, measurements satisfying the spectral version of the Born rule (i.e., projective measurements) are almost always idealizations. They are realistic only under special circumstances. Examples include two detection elements behind polarization filters perfectly polarizing in two orthogonal directions, or the arrangement in an idealized Stern–Gerlach experiment for spin measurement. Most measurements, and in particular all measurements of quantities with a continuous spectrum, are not projective.

The orthogonality relations imply that $XP_{k}=x_{k}P_{k}$. Since, the $P_{k}$ sum up to 1, any ψ∈H can be decomposed into a sum $\psi =\sum_{k}P_{k}\psi =\sum_{k}\psi_{k}$ of vectors $\psi_{k}:=P_{k}\psi$ satisfying the equation $X\psi_{k}=XP_{k}\psi =x_{k}P_{k}\psi =x_{k}\psi_{k}$. Therefore, each $\psi_{k}$ (if nonzero) is an eigenvector of *X* (or of each component of *X* in case the $x_{k}$ are not just numbers) corresponding to the eigenvalue $x_{k}$ of *X*. Since $P_{k}^{2}=P_{k}=P_{k}^{*}$, the $P_{k}$ are orthogonal projectors to the eigenspaces of the $x_{k}$.

When the $x_{k}$ are numbers, this implies that *X* is an operator with a finite spectrum. Moreover, *X* and $X^{*}$ commute; i.e., *X* is a normal operator, and in case the $x_{k}$ are real numbers, a Hermitian, self-adjoint operator. This is the setting traditionally assumed from the outset. When the $x_{k}$ are not numbers, our analysis implies that the components of *X* are mutually commuting normal operators with a finite joint spectrum, and if all $x_{k}$ only have real components, the components of *X* are Hermitian, self-adjoint operators. Thus, the projective setting is quite limited with respect to the kind of quantities that it can represent.

For projective measurements, (9) implies

$$f\left(X^{*},X\right)=\sum_{k}f\left({\bar{x}}_{k},x_{k}\right)P_{k}$$

for all functions *f* for which the right-hand side is defined. Therefore, the modified scale $f(x^{*},x)$ measures $f(X^{*},X)$, as we are accustomed from classical measurements, and defines a projective measurement of it. But when the components of *X* are not normal or do not commute, this relation does not hold.

Thus, we recover the traditional spectral setting as a consequence of the general approach, restricted to the special case where the components of *X* are commuting self-adjoint (or at least normal) operators, and hence have a joint spectral resolution with real (or complex) eigenvalues $x_{k}$, and the $P_{k}$ are the projection operators to the eigenspaces of *X*.

### 3.4. The Domain of Validity of Various Forms of Born Rule

It is well established that quantum mechanics applies universally to all physical scales, not only in the microscopic domain. This implies that the Born rule cannot apply to arbitrary physical measurements. It does not cover the multitude of situations where typically only a single measurement of a observable quantity is made. In particular, the Born rule does not apply to typical macroscopic measurements, whose essentially deterministic predictions are derived from quantum statistical mechanics.

Many other things physicists measure have no simple interpretation in terms of the Born rule. Often, many approximate computations are involved before raw observations lead to measurement results. Other examples include spectral lines and widths, life times of unstable particles, scattering cross-sections, or chemical reaction rates.

Thus, we now address the task of describing the domain of validity of the various formulations of the Born rule, delineating the class of measurements for which they gives a valid description.

#### 3.4.1. Invalidity of the Objective Forms

As discussed in Section 2.3, the objective forms (BR-OSc) and (BR-OE) of the Born rule are untenable, since the lack of commutativity of the operator algebra leads to the impossibility of assigning a joint probability distribution to position and momentum (and to other non-commuting observables).

Peskin & Schroeder [67] appeal on p. 104 to the objective expectation form (BR-OE) of the Born rule by considering *“the probability for the initial state to scatter and become a final state of n particles whose momenta lie in a small region”*. But this is the sloppiness that adherents of *shut-up-and-calculate* allow themselves, and indeed, they refer to measurement on the page before.

#### 3.4.2. Domain of Validity of the Measured Scattering Form

Born [5] had already mentioned in 1926 that the objective scattering form (BR-OSc) of the Born rule is applicable only in the absence of degeneracy. The same holds for the measured scattering form (BR-MSc). Under this restriction, the measured scattering form (BR-MSc) of the Born rule is impeccable and today remains the basis of the interpretation of S-matrix elements calculated in quantum mechanics and quantum field theory.

The additional rule that, in the degenerate case, one has to sum or integrate over a complete basis of out-states in the eigenspace of the joint spectrum of the asymptotically conserved quantities allows the correct interpretation of arbitrary scattering processes.

The quantum field theory book by Weinberg [68] pays, in his (2.1.7) on p. 50, lip service to the universal form (BR-US) of Born’s rule. But the only place in the book where Born’s rule is actually used is on p. 135: *“The probability of a multi-particle system, which is in a state α before the interaction is turned on, is found in a state β after the interaction is turned off, is”* his formula (3.4.7). The wording ‘is found’ shows that the measured scattering form (BR-MSc) is employed to obtain the formula (3.4.11) for the transition rates in scattering processes. Indeed, quantum field theory always predicts transition *rates*, not transition probabilities. In this context, it is interesting to note how Weinberg sweeps (on p. 134) the associated measurement problem under the carpet: *“with the excuse that (as far as I know) no interesting open problems in physics hinge on getting the fine points right regarding these matters”*.

#### 3.4.3. Domain of Validity of the Finite and the Discrete Form

On the other hand, the finite form (BR-FS) of the Born rule needs four conditions for its validity. It is valid precisely for measuring quantities

1. with only discrete spectrum,
2. measured over and over again in identical states (to make sense of the probabilities), where
3. the difference of adjacent eigenvalues is significantly larger than the measurement resolution, and where
4. the measured value is adjusted to exactly match the spectrum, which must be known exactly prior to the measurement.

These conditions are satisfied for measurements in the form of clicks, flashes, or events (particle tracks) in scattering experiments, and perhaps only then.

That these requirements are needed is due to the insistence of the spectral formulations that only exact eigenvalues are measured, a condition violated in many quantum measurement situations.

- A measurement of the mass of a relativistic particle with 4-momentum *p* never yields an exact eigenvalue of the mass operator $M:=\sqrt{p^{2}}$. Indeed, the masses of most particles are only inaccurately known.
- Energy measurements of a system at energies below the dissociation threshold (i.e., where the spectrum of the Hamiltonian *H*, the associated quantity, is discrete), almost never yield an exact eigenvalue of *H*, as the discrete form of the Born rule requires. Indeed, the energy levels of most realistic quantum systems are only inaccurately known. For example, nobody knows the exact value of the Lamb shift, a difference in eigenvalues of the Hamiltonian of the hydrogen atom; the first reasonably precise measurement was even worth a Nobel prize (1955 for Willis Lamb).
- The discrete part of the spectrum of a composite system is usually very narrowly spaced and precise energy levels are known only for the simplest systems, in the simplest approximations. Thus, the Born rule does not apply to the total energy of a composite system, according to Dirac one of the key quantities in quantum physics. In particular, the Born rule cannot be used to justify the canonical ensemble formalism of statistical mechanics; it can at best motivate it.
- Real measurements usually produce numbers that are themselves subject to uncertainty (NIST [69]), and rarely the exact numbers that the discrete form (BR-DS) of the Born rule requires. This implies that the spectral form of the Born rule paints an inadequate, idealized picture whenever eigenvalues are only approximately known and must therefore be inferred experimentally. For example, a **Stern–Gerlach experiment** measures (according to the common textbook story) the eigenvalues of the spin operator $L_{3}=\;\hbar \sigma_{3}$ (with angular momentum units), where ħ is Planck’s constant. The eigenvalues are ±ħ/2. Taking the spectral form of his rule literally, Born could have deduced in 1927 from the Stern–Gerlach experiment the exact value of Planck’s constant! But the original Stern–Gerlach experiment produced on the screen only two overlapping lips of silver, from which one cannot obtain an accurate value for ħ/2. Indeed, Busch et al. ([17], in Example 1, p. 7) write *"The following ‘laboratory report’ of the historic Stern-Gerlach experiment stands quite in contrast to the usual textbook ‘caricatures’. A beam of silver atoms, produced in a furnace, is directed through an inhomogeneous magnetic field, eventually impinging on a glass plate. […] Only visual measurements through a microscope were made. No statistics on the distributions were made, nor did one obtain ‘two spots’ as is stated in some texts. The beam was clearly split into distinguishable but not disjoint beams."*

#### 3.4.4. Domain of Validity of the Universal Form

The universal form of the (BR-US) Born rule inherits all limitations of the discrete form, but has additional limitations.

- The joint measurements of quantities represented by operators that do not commute cannot even be formulated in the textbook setting of projective measurements. Thus, the Born rule in its universal form does not apply, and one needs a quantum measure (POVM) that is not projective to model the measurement. For example, this applies for a simultaneous low-resolution measurement of position and momentum by inferring it from the trace of a particle in a cloud chamber.
- As first observed by Heisenberg ([70], p. 25), the Born rule implies a tiny but positive probability that an electron bound to an atom will be detected light years away from the atom: *“The result is more remarkable that it seems at first. For it is known that $\psi^{*}\psi$ decreases exponentially with increasing distance. Hence there is always a positive probability for finding the electron very far from the nucleus.”*
  (German original: *“Das Resultat ist aber merkwürdiger, als es im ersten Augenblick den Anschein hat. Bekanntlich nimmt $\psi^{*}\psi$ exponentiell mit wachsendem Abstand vom Atomkern ab. Also besteht immer noch eine endliche Wahrscheinlichkeit dafür, das Elektron in sehr weitem Abstand vom Atomkern zu finden.”*).
  Therefore, ${\left|\psi \left(x\right)\right|}^{2}$ is unlikely to be the *exact* probability density for being detected at *x*, as (BR-US) would require. This indicates that, for observables with continuous spectrum, (BR-US) must be an idealization.

#### 3.4.5. Domain of Validity of the Condensed Form

The statistical interpretation based on the Born rule in the condensed form (BR-C) and in the POVM form (BR-POVM) are derivable from the DRP, and give a correct account of the actual experimental situation. They apply, without idealization, to the results of arbitrary quantum measurements in the sense of the DRP. For a large number of examples see Neumaier & Westra ([8], Section 6.2).

However, (BR-C) and (BR-POVM) still have the same limitations as the DRP, and cannot be applied to measurements of the kind discussed at the beginning of this subsection, such as macroscopic measurements or the measurements of spectral lines. For example, spectroscopy-based high-precision measurements such as the gyromagnetic ratio of the electron are not quantum measurements but measurements of numerical parameters in specific quantum models; see the detailed discussion in Neumaier & Westra ([8], Section 5.2).

### 3.5. What Is Missing in the Foundations?

At every steadily progressing moment, Nature somehow produces from *what has been up to now* (defined by what is observable, at least in principle) that *what has been up to a while later*.

Physics is supposed to quantitatively describe and understand this in terms of physical theories and models, with which predictions can be made, and in terms of experiments that evaluate some of the details of *what has been up to now*, with which such predictions can be prompted, verified, or refuted. But Nature proceeds without caring about our physical models and the knowledge built into them, and has done so long before the first living observers existed.

The fundamental physical state λ(t) is a complete description of the (to us unknown) collection of everything that *has been up to time t*. The λ(t) for all *t* fully describe *what is*. This defines an effective ontology based on what is observable in principle.

The (to us unknown) fundamental dynamical law of physics determines how λ(t) for $t>t_{0}$ is related to $\lambda (t_{0})$.

Our physical models and the knowledge built into them reflect our collective attempts to distill from *what has been up to now* an approximate picture of the fundamental physical state and the fundamental dynamical law that allows us to predict a good approximation to *what is*.

Curiously, at present, our physical models are not in terms of what is (i.e., of approximations to λ(t), part of which is observed) but in terms of states ψ or ρ and observables *X* only indirectly related to what is. This indirect relation between the quantum theory of states and observables and reality is given by one of the versions of the Born rule.

The above notion of *what is* must be distinguished from the theoretical ontology of certain interpretations of quantum mechanics: In the Copenhagen interpretation, *what is* is the classical world, delineated from the quantum world by a movable Heisenberg cut. In the consistent histories approach (Griffiths [71]), *what is* is the single history up to our present, which is just one of an uncountable collection of consistent histories, to which theory only contributes uncheckable probability assignments to each. In many-world interpretations, a preferred ontological status is given to a postulated universal wave function, a theoretical figment of abstraction, whereas *what is* (in the present sense) is degraded to a particular world amid infinitely many worlds, namely the single distinguished world that is our actual past, and from which we have to learn everything we can know about Nature, independently of any weight one might give to this world or to other worlds. In Bohmian mechanics, infinitely precise positions and the exact wave function are declared to have a preferred ontological status—a poor, theoretical substitute for the colorful *what is* that we experience. In QBism (Fuchs ([72], Section 3)), *what is* (in the present sense) is degraded to the world of a metaphysical society of agents governed by moral imperatives of how they should bet. In each case, nothing real (in the sense of our *what is*) is in the quantum theory (of states and observables) itself, a state of affairs criticized by Einstein throughout his life.

The Born rule depends on a notion of measurement that has no formal counterpart in quantum mechanical models. This means that we are currently unable to theoretically explain which formal quantum models of pieces of matter describe a quantum detector. The theoretical explanation for why certain objects are quantum detectors constitutes the so-called quantum measurement problem.

If we accept the DRP, the problem is to give mathematical conditions for certain individual quantum objects described by microscopic quantum mechanics that guarantee that the DRP holds for a suitable mathematical definition of what it means for the quantum model to respond at a particular time. This is a nontrivial unsolved problem in the statistical mechanics of detector properties.

In 1962, Daneri et al. [73] initiated the discussion of quantum detectors by means of quantum statistical mechanics. However, they assumed the objective scattering form (BR-Sc) of the Born rule: *“the intermediate state (depending on the direction in which the particle is scattered) is the state in which ions are created inside the k-th counter”* (p. 308). In addition, they assumed that the detector is dissipative (p. 307). The latter (but not some version of the Born rule) could be avoided by using a metastable quantum device coupled to a heat bath with a continuous frequency spectrum, along the lines of Caldeira & Leggett [74].

The work by Allahverdyan et al. [59] goes some way towards resolving the problem. But they have to equate, at certain points, the formal probabilities of statistical mechanics (cf. Section 1.3) with measured probabilities, thus silently invoking the Born rule. This is done at the beginning of their Section 11, where they say that *“the statistical interpretation of quantum mechanics emphasizes the idea that this theory, whether it deals with pure or mixed states, does not govern individual systems but statistical ensembles of systems”*. But it describes statistical ensembles only if the formal probabilities are taken to be observed frequentist probabilities, which requires Born’s rule. Thus, the derivation is circular. The problem is still present in their recent paper ([75], p. 254), since they assume the maximum entropy principle, which (as discussed above in Section 1.3) requires the condensed form (BR-C) of Born’s rule. Moreover, to go from an ensemble to an individual case, they need an additional characterization of a quantum measuring apparatus *M*, somewhat similar to the DRP of SubSection 3.1, called Postulate P. This postulate, *“although intuitive, is not a consequence of the mere quantum principles and has a macroscopic nature. We will accept its inclusion in quantum theory as a postulate (motivated by the macroscopic size of M)”*. In effect, they show that (BR-C) assumed for quantum statistical mechanics, together with their new Postulate P, implies that the measurement device they analyzed responds as the Born rule requires. Thus, they reduced the measurement problem to their Postulate P.

The decoherent history approach of Gell-Mann & Hartle [76] for the derivation of classical probability suffers from the problem of having to reinterpret formal smeared Wigner functions as classical probability densities (cf. my introduction to Section 3), whose statistical interpretation needs (BR-C). Indeed, they assert on p. 3378 that something is missing: *“we may begin to tackle the deep problem of introducing individuality into quantum mechanics. Actual alternative histories deal, of course, in large part with individual objects such as our galaxy, the Sun, the Earth, biological organisms on the Earth, and so forth. Yet discussions of quantum mechanics up to now have typically treated such individual objects only as external systems, labeled as ‘observers’ and ‘pieces of apparatus’.”*

The main problem behind these difficulties is the need to find a rigorous derivation of a dissipative classical dynamics for a single pointer (or switch), without silent recourse to the Born rule. A customary way to proceed (following Möhring & Smilansky [77]) is to start with the influence functional approach of Feynman & Vernon [78]. However, this approach has the Born rule built in—namely into the derivation (p. 122) of the basic functional integral representation of the transition probabilities.

A solution of the measurement problem is indeed impossible unless one has a precise mathematical definition of when and how a mathematical model of a single quantum detector produces a definite response. E.g., Busch & Lahti ([79], p. 375) write *“There is no consistent quantum measurement theory, unless an appropriate reinterpretation of what it means for an observable to have a definite value can be found.”.* This is the reason why, 100 years after the inception of quantum mechanics in terms of states and observables, the quantum measurement problem is still unsolved. It is the missing piece in the foundations of quantum mechanics. 

## Data Availability

The original contributions presented in this study are included in the article. Further inquiries can be directed to the corresponding author.

## References

[1] Jeans J.H. (1924). Report on Radiation and the Quantum Theory.

[2] Bohr B., Kramers H.A., Slater J.C. (1924). Über die Quantentheorie der Strahlung. Z. Phys..

[3] Nordheim L. (1926). Zur Theorie der Anregung von Atomen durch Stösse. Z. Phys..

[4] Schrödinger E. (1926). Quantisierung als Eigenwertproblem (Vierte Miteilung). Ann.Phys..

[5] Born M. (1926). Quantenmechanik der Stossvorgänge. Z. Phys..

[6] (1954). Nobel Prize in Physics. https://www.nobelprize.org/prizes/physics/1954/summary/.

[7] Neumaier A. (2019). Coherent Quantum Physics.

[8] Neumaier A., Westra D. (2024). Algebraic Quantum Physics, Volume 1: Quantum Mechanics via Lie Algebras.

[9] Neumaier A. (2019). Born’s Rule and Measurement, Unpublished Manuscript. https://arxiv.org/abs/1912.09906.

[10] Neumaier A. (2019). Foundations of quantum mechanics I. A critique of the tradition. arXiv.

[11] Neumaier A. (2021). Quantum tomography explains quantum mechanics. arXiv.

[12] Wikipedia, Born Rule. https://en.wikipedia.org/wiki/Born_rule.

[13] Davies E.B., Lewis J.T. (1970). An operational approach to quantum probability. Commun. Math. Phys..

[14] Ali S.T., Emch G.G. (1974). Fuzzy observables in quantum mechanics. J. Math. Phys..

[15] Wikipedia, Positive Operator-Valued Measure (POVM). https://en.wikipedia.org/wiki/Positive_operator-valued_measure.

[16] Nielsen M.A., Chuang I.L. (2011). Quantum Computation and Quantum Information: 10th Anniversary Edition.

[17] Busch P., Grabowski M., Lahti P. (1995). Operational Quantum Physics.

[18] Englert B.G. (2013). On quantum theory. Eur. Phys. J. D.

[19] Brandt H.E. (1999). Positive operator valued measure in quantum information processing. Am. J. Phys..

[20] D’Ariano G.M., Paris M.G.A., Sacchi M.F. (2003). Quantum tomography. Adv. Imaging Electron. Phys..

[21] Fuchs C.A., Peres A. (1996). Quantum-state disturbance versus information gain: Uncertainty relations for quantum information. Phys. Rev. A.

[22] Naimark M.A. (1943). On a Representation of Additive Operator Set Functions. Dokl. Akad. Nauk SSSR.

[23] Balian R., Balazs N.L. (1987). Equiprobability, inference, and entropy in quantum theory. Ann. Phys..

[24] Heisenberg W. (1927). Über den anschaulichen Inhalt der quantentheoretischen Kinematik und Mechanik. Z. Phys..

[25] Dirac P.A.M. (1930). The Principles of Quantum Mechanics.

[26] von Neumann J. (1928). Wahrscheinlichkeitstheoretischer Aufbau der Quantenmechanik. Nachrichten Ges. Wiss. GöTtingen-Math.-Phys. Kl..

[27] Bauer E. (1934). Théorie quantique de la valence. Les liaisons homéopolaires. Bull. Soc. Chim. Fr. Mém..

[28] Ziegler F. Answer to Who Coined the Term “Born’s Rule”? History of Science and Mathematics, Q&A Site, 1 July 2018. https://hsm.stackexchange.com/a/7488/3915.

[29] Jordan P. (1927). Über eine neue Begründung der Quantenmechanik. Z. Phys..

[30] Wessels L. (1980). What was Born’s statistical interpretation?. PSA: Proceedings of the Biennial Meeting of the Philosophy of Science Association.

[31] Mehra J., Rechenberg H. (2000). The Historical Development of Quantum Theory, Volume 6: The Completion of Quantum Mechanics, 1926–1941, Part 1: The Probability Interpretation and the Statistical Transformation Theory, the Physical Interpretation, and the Empirical and Mathematical Foundations of Quantum Mechanics 1926–1932.

[32] Bacciagaluppi G., Freire O., Bacciagaluppi G., Darrigol O., Hartz T., Joas C., Kojevnikov A., Pessoa O. (2022). The Statistical Interpretation: Born, Heisenberg, and von Neumann. The Oxford Handbook of the History of Quantum Interpretations.

[33] Born M. (1926). Zur Quantenmechanik der Stossvorgänge. Z. Phys..

[34] Wheeler J.A. (1937). On the Mathematical Description of Light Nuclei by the Method of Resonating Group Structure. Phys. Rev..

[35] Dirac P. (1927). Über die Quantenmechanik der Stossvorgänge. Z. Phys..

[36] Born M. (1927). Das Adiabatenprinzip in der Quantenmechanik. Z. Phys..

[37] de Broglie L. (1929). Einführung in Die Wellenmechanik.

[38] Jordan K.F. (1898). Grundriss der Physik nach dem Neuesten Stande der Wissenschaft.

[39] Kramers H.A., Heisenberg W. (1925). Über die Streuung von Strahlung durch Atome. Z. Phys..

[40] Heisenberg W. (1946). Der unanschauliche Quantensprung. Phys. Blätter.

[41] von Meyenn K. (2011). Eine Entdeckung von ganz Ausserordentlicher Tragweite: Schródingers Briefwechsel zur Wellenmechanik und zum Katzenparadoxon.

[42] Fowler R.H. (1926). General Forms of Statistical Mechanics with Special Reference to the Requirements of the New Quantum Mechanics. Proc. R. Soc. Lond. A.

[43] Pauli W. (1979). Wissenschaftlicher Briefwechsel mit Bohr, Einstein, Heisenberg u.a., Band I: 1919–1929.

[44] Pauli W. (1927). Über Gasentartung und Paramagnetismus. Z. Phys..

[45] von Neumann J. (1928). Mathematische Begründung der Quantenmechanik. Nachrichten Ges. Wiss. GöTtingen-Math.-Phys. Kl..

[46] Landau L. (1927). Das Dämpfungsproblem in der Wellenmechanik. Z. Phys..

[47] Weyl H. (1927). Quantenmechanik und Gruppentheorie. Z. Phys..

[48] Lennard-Jones J.E. (1931). The Principles of Quantum Mechanics. By P.A.M. Dirac. Math. Gaz..

[49] Pauli W. (1959). Albert Einstein in der Entwicklung der Physik. Phys. Blätter.

[50] Jaynes E.T. (1957). Information theory and statistical mechanics. Phys. Rev..

[51] Dirac P.A.M. (1947). The Principles of Quantum Mechanics.

[52] Landau L.D., Lifshitz E.M. (1977). Course of Theoretical Physics, Volume 3: Quantum Mechanics.

[53] Chuang I.L., Nielsen M.A. (1997). Prescription for experimental determination of the dynamics of a quantum black box. J. Mod. Opt..

[54] Mohseni M., Rezakhani A.T., Lidar D.A. (2008). Quantum-process tomography: Resource analysis of different strategies. Phys. Rev. A.

[55] Schlosshauer M. (2007). Decoherence and the Quantum-To-Classical Transition.

[56] Fine A. (1973). Probability and the interpretation of quantum mechanics. Br. J. Philos. Sci..

[57] Gilton M.J. (2016). Whence the eigenstate-eigenvalue link?. Stud. Hist. Philos. Sci. B.

[58] Schlosshauer M., Fine A. (2005). On Zurek’s derivation of the Born rule. Found. Phys..

[59] Allahverdyan A.E., Balian R., Nieuwenhuizen T.M. (2013). Understanding quantum measurement from the solution of dynamical models. Phys. Rep..

[60] Vaidman L., Svozil K. (2020). Derivations of the Born rule. Quantum, Probability, Logic: The Work and Influence of Itamar Pitowsky.

[61] Schonfeld J.F. (2021). The first droplet in a cloud chamber track. Found. Phys..

[62] Langer J.S. (1969). Statistical theory of the decay of metastable states. Ann. Phys..

[63] Lombardo F.C., Mazzitelli F.D., Rivers R.J. (2003). Decoherence in field theory: General couplings and slow quenches. Nucl. Phys. B.

[64] Chanda N., Bhattacharyya R. (2021). Emergence of the Born rule in strongly driven dissipative systems. Phys. Rev. A.

[65] Aharonov Y., Shushi T. (2024). Improving the proof of the Born rule using a physical requirement on the dynamics of quantum particles. Quantum Stud. Math. Found..

[66] Kolmogorov A. (1956). Foundations of the Theory of Probability.

[67] Peskin M.E., Schroeder D.V. (1996). An Introduction to Quantum Field Theory.

[68] Weinberg S. (1995). The Quantum Theory of Fields, Volume 1, Foundations.

[69] NIST International and U.S. Perspectives on Measurement Uncertainty.

[70] Heisenberg W. (1930). Die Physikalischen Prinzipien der Quantentheorie.

[71] Griffiths R.B. (2024). The Consistent Histories Approach to Quantum Mechanics; Stanford Encyclopedia of Philosophy. https://plato.stanford.edu/ENTRIES/qm-consistent-histories/.

[72] Fuchs C.A. (2023). QBism, where next?. arXiv.

[73] Daneri A., Loinger A., Prosperi G.M. (1962). Quantum theory of measurement and ergodicity conditions. Nucl. Phys..

[74] Caldeira A.O., Leggett A.J. (1983). Quantum tunnelling in a dissipative system. Ann. Phys..

[75] Allahverdyan A.E., Balian R., Nieuwenhuizen T.M. (2024). Teaching ideal quantum measurement, from dynamics to interpretation. C. R. Phys..

[76] Gell-Mann M., Hartle J.B. (1993). Classical equations for quantum systems. Phys. Rev. D.

[77] Möhring K., Smilansky U. (1980). A semi-classical treatment of dissipative processes based on Feynman’s influence functional method. Nucl. Phys. A.

[78] Feynman R.P., Vernon F.L. (1963). The theory of a general quantum system interacting with a linear dissipative system. Ann. Phys..

[79] Busch P., Lahti P., Greenberger D., Hentschel K., Weinert F. (2009). Measurement theory. Compendium of Quantum Physics.

